# Functional proteomics of barley and barley chloroplasts – strategies, methods and perspectives

**DOI:** 10.3389/fpls.2013.00052

**Published:** 2013-03-18

**Authors:** Jørgen Petersen, Adelina Rogowska-Wrzesinska, Ole N. Jensen

**Affiliations:** Department of Biochemistry and Molecular Biology, University of Southern DenmarkOdense, Denmark

**Keywords:** barley, *Hordeum vulgare*, proteomics, chloroplast, mass spectrometry, 2D gel electrophoresis

## Abstract

Barley (*Hordeum vulgare*) is an important cereal grain that is used in a range of products for animal and human consumption. Crop yield and seed quality has been optimized during decades by plant breeding programs supported by biotechnology and molecular biology techniques. The recently completed whole-genome sequencing of barley revealed approximately 26,100 open reading frames, which provides a foundation for detailed molecular studies of barley by functional genomics and proteomics approaches. Such studies will provide further insights into the mechanisms of, for example, drought and stress tolerance, micronutrient utilization, and photosynthesis in barley. In the present review we present the current state of proteomics research for investigations of barley chloroplasts, i.e., the organelle that contain the photosynthetic apparatus in the plant. We describe several different proteomics strategies and discuss their applications in characterization of the barley chloroplast as well as future perspectives for functional proteomics in barley research.

## INTRODUCTION

Barley (*Hordeum vulgare*) is one of the earliest domesticated cereals and it is the fourth most important crop world-wide in terms of total dry production, only exceeded by maize, rice, and wheat. Barley is mainly used in the brewing industry and as animal feed, but in certain areas of the world it is an important food source for humans (Schulte et al., 2009). The increasing demand for food due to the growing world population has propelled the implementation of plant breeding programs and biomolecular plant research to improve sustainable crop production. Prioritized areas include research in plant resistance to abiotic stress such as soil salinity, temperature, drought, nutrient uptake (Saeed et al., 2012), and biotic stress caused by other living organisms and pathogens (Dreher and Callis, 2007). Barley is by nature diploid, has a low chromosome number (2*n* = 14) and a large genome size (5.1 Gb), is easy to cross-breed and is able to grow under various climatic conditions. These abilities and the fact that barley is an extremely important crop makes it desirable to identify genes responsible for specific beneficial traits in order to improve crop production and sustainability (Saisho and Takeda, 2011). The recently completed whole-genome sequencing of the barley genome (Mayer et al., 2012), gave rise to several interesting observations. A total of 26,159 high confidence genes with gene-family similarity to other plant genomes, and 53,220 genes with lack of homology denoted low confidence genes were identified. By comparison to *Arabidopsis thaliana*, the barley genome was estimated to encompass 30,400 genes. RNA sequencing data indicated extensive alternative splicing of the coding regions of the high confidence genes (Mayer et al., 2012), this adds to protein diversity and may play a role in protein regulation and gene expression (Syed et al., 2012). These data opens new opportunities for pursuing in-depth studies of barley biology by using genomics, transcriptomics, metabolomics, and proteomics approaches.

The chloroplast is one of the specialized plastids in the plant cell and it conducts important processes such as photosynthesis and biosynthesis of amino acids, starch, and vitamins. The chloroplast contains its own genome, but most of the estimated 2000–3000 chloroplast proteins are encoded by the nuclear genome. Targeting of proteins to the chloroplast often requires N-terminal pre-sequences called chloroplast transit peptides (cTPs), which to some extend can be predicted from the genome by using computational methods such as chloroP, targeP, WoLF PSORT, iPSORT, predotar, or Protein Prowler (Emanuelsson et al., 1999; Bannai et al., 2002; Small et al., 2004; Boden and Hawkins, 2005; Horton et al., 2007).

Functional proteomics is a rapidly evolving scientific discipline that is driven by advancements in a series of bioanalytical and computational technologies to enable increasingly detailed studies of complex protein mixtures derived from cells, tissues, and organisms (Aebersold and Mann, 2003; Cravatt et al., 2007; Bensimon et al., 2012). The main methods used in proteomics are: (1) protein and peptide separation techniques; (2) mass spectrometry; (3) biological sequence databases and computational query tools (summarized in **Boxes 1** and **2**).

Proteomics technologies are now extensively used in plant biology, particularly in studies of the model plants and the most important food crops (Jorrin et al., 2007). Proteomics, i.e., the systematic study and characterization of proteins in a cell type, tissue, or a whole organism, encompasses the mapping of protein composition and abundance, protein interactions and protein localization, as well as dynamic events in protein regulatory networks, including signaling mechanisms, metabolism, and transcription (de Hoog and Mann, 2004). A majority of such studies in plants were carried out in *A. thaliana* and rice where completely sequenced genomes are available (Kaul et al., 2000; Goff et al., 2002). Proteome analysis of plant organelles, including chloroplasts, have been reported (Kleffmann et al., 2004). For example, proteomics strategies were used to elucidate the influence of various biotic and abiotic stresses on chloroplasts proteins.

The recently completed sequencing of the barley genome now provides a foundation for more detailed functional proteomics studies of barley biology. We therefore foresee an increased effort in barley proteomics using state-of-the-art mass spectrometry based strategies for qualitative and quantitative characterization of barley proteins, organelles and regulatory networks. Proteomics will likely play a major role in further improvements of barley cultivars, e.g., by identifying the underlying mechanisms of biotic and abiotic stress. In the following sections we provide an overview of proteomics strategies and techniques and the current state of barley chloroplast proteomics.

**BOX 1. Mass spectrometry.**

Mass spectrometry enables unambiguous identification of proteins by accurate mass measurements of gas-phase protein and peptide ions and peptide fragment ions. Mass spectrometers using matrix-assisted laser desorption ionization (MALDI) are preferred for simple peptide mixtures derived by in-gel digestion of proteins obtained from 2D gel spots (Gevaert and Vandekerckhove, 2000). Electrospray ionization (ESI) mass spectrometers are frequently interfaced directly to nanoliter-flow HPLC systems, thereby providing separation, mass determination, and amino acid sequencing in one analytical setup (LC-MS/MS) (Aebersold and Mann, 2003). Besides being able to identify thousands of proteins in one single LC-MS/MS analysis, modern proteomics workflows also provides rather accurate protein quantification and capability to identify PTMs (Larsen et al., 2006; van Bentem et al., 2006; Ytterberg and Jensen, 2010; Mithoe and Menke, 2011). These features make MALDI and ESI mass spectrometry indispensable in proteomics research for the characterization and quantification of complex protein mixtures.

**Box 2. Quantitative proteomics.**

2D gel electrophoresis is the preferred method for comparative quantitative proteomics in studies of organisms for which only incomplete gene annotation is available, e.g., for carrots and cabbage (Nawrocki et al., 2011). The advantage of using 2D gel electrophoresis is the one spot – one protein premise that makes it relative easy to make sequence homology searches, de-novo sequencing of fragmented peptides or protein isoform characterization (Jacob and Turck, 2008; Moller et al., 2011b).

Mass spectrometry driven quantitative proteomics methods can be categorized into “label-free” approaches based on peptide intensity or peptide counting and “stable isotope labeling” methods where proteins and/or peptides are metabolically or chemically encoded by heavy stable isotopes of, e.g., carbon, nitrogen, and oxygen (13-C, 15-N, 18-O; Ong and Mann, 2005; Thelen and Peck, 2007; Bantscheff et al., 2012). Commonly used metabolic labeling methods in plant proteomics include stable isotope labeling by 15-N (Nelson et al., 2007; Bindschedler et al., 2008; Gouw et al., 2008) and by amino acids in cell culture [stable isotope labeling by amino acids in cell culture (SILAC); Ong et al., 2002], although the latter is not easily implemented in plants due to their amino acid metabolism (Gruhler et al., 2005). Chemical methods for stable isotope labeling are generically applicable in plant proteomics and include iTRAQ (Ross et al., 2004; Wiese et al., 2007) and isotope-coded protein labeling (ICPL; Schmidt et al., 2005). Examples include phosphoproteomics (Jones et al., 2006; Melo-Braga et al., 2012), global protein regulation in response to stress (Neilson et al., 2011; Abdalla and Rafudeen, 2012) or as a consequence of genotypic differences (Chen et al., 2009; Ng et al., 2012).

The advantage of label-free approaches is that they are rather straightforward to implement, however, their robustness and accuracy relies on multiple replicate runs and comparative data analysis is often rather complex. Nevertheless, recent improvements in software and statistics for label-free proteomics make this a very attractive approach. The main advantages of stable isotope labeling techniques are their accuracy of quantification and the ability to perform multiplex experiments. iTRAQ allows up to eight-plex analysis in one LC-MS/MS experiment (Bantscheff et al., 2007; Pottiez et al., 2012).

## GENERAL CONSIDERATIONS AND PROTEOMICS STRATEGIES

Several factors affect the outcome of a proteomics experiment, and need to be included in the experimental planning phase, like for example proteome complexity and protein concentration (summarized in **Box 3**). This section covers two classical proteomics strategies and highlights things to consider before starting a chloroplast-targeted proteomics experiment.

**BOX 3. Proteome complexity and protein concentrations.**

Due to the high complexity and wide concentration range of proteins within proteomes, large scale proteome analysis is often executed at the sub-proteome level (James, 1997; Kuntz and Rolland, 2012) where specific cellular or tissue fractions are isolated and analyzed. For example, enrichment strategies can be used to isolate sub-proteome consisting of, e.g., kinases, or proteins containing specific modifications (e.g., phosphorylation or glycosylation), body or tissue fluids (e.g., sap) or organelles such as cell nuclei, mitochondria, Golgi apparatus, or chloroplasts. The need for fractionation into sub-proteomes becomes obvious when considering that the potential number of different proteins from a single genome coding for 20,000–30,000 genes, might be as high as 200,000–2 million when considering genomic recombination, splice variants, differential initiation/termination of transcripts and protein processing and covalent modifications (Ayoubi and Van De Ven, 1996; Lander et al., 2001). In addition, the concentration ranges of proteins in eukaryotic cells typically span five–six orders of magnitude and in some sub-proteomes as high as 10 orders of magnitude. In some plants it has been estimated that RuBisCO makes up 40% of the total protein content, making the stroma in the chloroplast a very challenging protein matrix to analyze (Patterson and Aebersold, 2003; Bindschedler and Cramer, 2011). By reducing protein complexity by sub-proteome fractionation it is possible to identify low abundant proteins in the proteome of an organism.

*Purification: *The first step toward success in organelle or sub proteomic experiment is the quality and purity of the sample. Contaminating proteins or unwanted cellular debris can obscure the results with respect to assignment of organelle specific proteins and their quantification (Agrawal et al., 2011). Highly purified chloroplasts or mitochondria can be obtained using a Percoll gradient centrifugation step (Neuburger et al., 1982; Aronsson and Jarvis, 2002; van Wijk, 2004; Millar et al., 2005). Endomembrane organelles such as Golgi apparatus, endoplasmic reticulum, vacuoles, and vesicles are more difficult to purify without cross-contamination from other organelles. Gentle rupture of the intact chloroplasts enables further purification of four sub-compartments (1) the inner and outer envelope membranes, (2) the stroma, (3) the thylakoid membrane, (4) the thylakoid lumen (Kieselbach et al., 1998, 2000; Peltier et al., 2000, 2006; Schubert et al., 2002; Ferro et al., 2003). The above mentioned extractions method were used for diverse plant species, and it is important to have in mind that protocols developed for a specific plant species, not necessarily works for other species. Typically intact chloroplasts are obtained using Percoll gradient centrifugation. This is by far the best way to obtain pure chloroplasts, but the yield is rather low. Less pure chloroplast can be obtained in high yields using low speed centrifugation. It is possible to obtain thylakoid, stroma, and envelope fractions using a sucrose gradient of osmotic shocked intact chloroplasts. Soluble luminal thylakoid proteins can be isolated from the thylakoid preparation using yeda press rupture of the membranes (Hall et al., 2011).

*How much material is needed?* It is possible to make quantitative proteomics experiments with less than 20μg of extracted protein. The number of identified proteins from such an experiment depends not only on the complexity and dynamics of the proteome but also the in-house instrumentation (Eriksson and Fenyo, 2010). In sub proteomic work the amount of starting material might exceed several grams to extract a few micro grams of a desired proteome. As an example, from 100 g of soil grown *A. thaliana* plants it is possible to extract approximately 1000 mg leaf protein, 100 mg thylakoid proteins, and only 0.4 mg envelope membrane protein (Froehlich et al., 2003).

*What buffers should I use?* There is no universal buffer composition to be used in proteomics experiments. Depending on the targeted tissue or sub-cellular compartment different protein extraction and sample preparation buffers are used (Fido et al., 2004; Mano et al., 2008). However, there are few universal rules that should be taken into consideration. Always add protease inhibitors, but be aware of the lifetime of the inhibitors, it might be short under certain conditions, or use strong denaturing buffers [e.g., 8 M urea or sodium dodecyl sulfate (SDS)] to inactivate potential proteolytic activity of enzymes present in the sample. Use metal chelating agents, e.g., ethylenediaminetetraacetic acid (EDTA) to trap free metal ions from the sample to prevent unwanted spontaneous protein oxidation – this is particularly important when working with organelles such as chloroplasts and mitochondria. Most buffers used in biological experiments contains components which are not compatible with liquid chromatography-mass spectrometry (LC-MS) but if the proteins are separated by polyacrylamide gel electrophoresis (PAGE) all buffers are allowed, due to the excellent washing ability of gel plugs. For non-gel based strategies some compounds such as detergents (SDS, Triton X-100, etc.) or ampholytes compromise nanoliter-flow LC or mass spectrometry and they need to be avoided or removed prior to analysis (Xiao et al., 2004; Yeung and Stanley, 2010).

*Can high abundant proteins be removed?* In photosynthetic tissue the predominant protein is the carbon fixation protein ribulose-1,5-bisphosphate carboxylase/oxygenase (Rubisco). In some cases more than 50% of the total leaf protein content consist of Rubisco (Metodiev and Demirevskakepova, 1992). Such highly abundant protein will hamper both gel and non-gel based proteome analysis because this highly abundant protein will obscure other proteins and suppress their detection. In gel based studies it will dominate the gel pattern eclipsing low abundant proteins with similar physico-chemical properties. In non-gel based peptides generated from this abundant protein will saturate high performance liquid chromatography (HPLC) columns and suppress the signal from lower abundant proteins. This problem can be partially solved by removing the highly abundant protein by fractionation, antibody based spin columns, or using the relative newly developed ProteoMiner beads (Boschetti and Righetti, 2008; Frohlich et al., 2012). Removal of highly abundant proteins can also result in removal of the associated low abundant proteins (Cellar et al., 2008; Krishnan and Natarajan, 2009). Another way to reduce the complexity and the dynamics of protein sample is to perform organelle or sub-organelle fractionation. Isolation of mitochondria or a thylakoid preparation from chloroplast will exclude the majority of Rubisco protein from the analysis.

*How to proceed after proteome extraction? *Proteins, both for gel and non-gel based strategies (see below) need to be digested into peptides prior to mass spectrometry analysis. The aim is to generate ionizable peptides in the mass range 700–2500 Da, which is the optimal range for most biological mass spectrometers. Disulfide bridges (Cys-Cys) in proteins are typical reduced and alkylated using dithiothreitol (DTT) and iodacetamide (IAA) prior to digestion. Denaturation of the proteins improves digestion efficiency, thus contributing to the overall protein identification rate. Proteins separated by SDS-PAGE are inherently denatured and are typically cut out of the gel, reduced, *S*-alkylated and digested by trypsin. This is a well-established “in-gel digestion” technique routinely used by most proteomics laboratories (Shevchenko et al., 1996, 2006). In solution based digestion is a more delicate procedure. Keeping the proteins in solution, denatured and available for trypsin digestion can be facilitated by buffers containing the commercially available surfactant**RapiGest, urea buffers or detergents such as sodium deoxycholate (SDC) that, in contrast to SDS can relatively easy be removed from the sample prior to the mass spectrometry analysis (Speers and Wu, 2007; Norrgran et al., 2009; Lin et al., 2012). In-solution digestion protocols where the digestion is performed within a spin filter device has become popular and is highly recommended for the digestion of protein amounts exceeding 100 μg. The filter enables washing of the sample and retention of large unwanted structures on the filter (Manza et al., 2005; Wisniewski et al., 2009).

*How do I evaluate the quality of the experiment*? Proteomics experiments often aim to detect differential regulated proteins between groups. This can be accomplished using a statistical test based on hypotheses about characteristics of both the biological samples that represent the population, and the variability of the technical measurements (Podwojski et al., 2012).

If possible, evaluate the protein extract by electrophoresis; this gives an overall picture of the extract. Non-gel based approaches can benefit using an internal spike-in protein standard. The protein standard is digested together with the extract, and by comparing sequence coverage and peptide intensities of the spiked-in standard among samples, the digest efficiency can be evaluated. This can be archived using selected reaction monitoring (SRM) or other label-free quantification methods. Absolute quantification can be archived using spiked-in peptides that act as internal standards (Gerber et al., 2003; Silva et al., 2006).

### PROTEOMICS STRATEGIES

**FIGURE 1:**
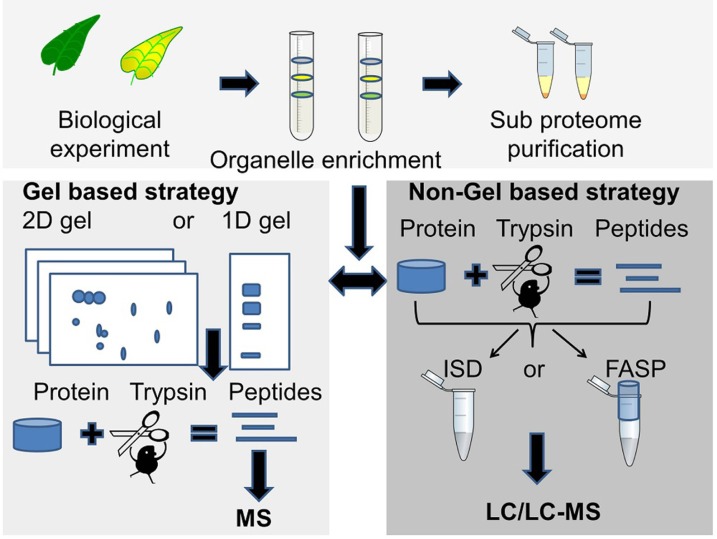
**Two commonly used proteomics strategies**. The gel based strategy (lower left panel), where protein bands or spots are cut out of the gel followed by trypsin digestion and MS analysis of peptides, and the MS based strategy (lower right panel) where the unseparated or partially fractionated protein sample is “in-solution digested” (ISD) with trypsin or digested in a spin filter [filter-aided sample preparation (FASP)] followed by LC/LC-MS/MS for peptide separation and sequencing.

The choice of proteomic strategy depends on several factors such as the overall aim of the proteomics experiment, protein sample complexity and protein amount, number of samples to analyze, mass spectrometry instrument considerations, sequence database availability and whether protein quantification is necessary (**Figure 1**).

2D gel electrophoresis is a separation technique that is based on isoelectric focusing of the proteins followed by separation of the proteins according to their molecular mass. It has been used in proteomics for more than 30 years. Although a number of its limitations have been recognized (reviewed in Issaq and Veenstra, 2008; Chevalier, 2010) it is an effective strategy for the separation and quantitation of intact protein mixtures, including protein isoforms and modified proteins. A variation of the classical denaturing 2D PAGE is blue native (BN) 2D PAGE (Reisinger and Eichacker, 2007). This technique has been used in several membrane proteins studies (Krause, 2006), and is also one of the preferred ways for characterization of protein complexes. Protein separated by electrophoresis are visualized by staining, isotope or fluorescent labeling (Patton, 2002). Often only the differential regulated proteins are selected for spot picking, protein digestion, and protein identification (Berth et al., 2007). The advantage using the 2D gel strategy is the one spot – one protein premise, which allows for relatively easy *de novo* annotation of peptide fragment spectra and homolog search.

The combination of SDS-PAGE and LC-MS is very efficient for proteome profiling. The combination is often called GeLC-MS/MS, and is excellent for proteome profiling due to the unbiased solubilization of all protein groups including membrane proteins. For quantitative measurements it can be used with metabolically incorporated stable isotopes, isobaric tags for relative and absolute quantitation (iTRAQ) and semi quantitative approaches such as spectral counting (Sachon et al., 2006; Wienkoop et al., 2006).

Recently, 2D LC-MS/MS strategies have become more widespread and robust. The orthogonality between the two LC separation dimensions is often obtained by using strong cation exchange chromatography (SCX) in the first dimension and reverse phase (RP) chromatography in the second dimension, separating the peptides according to charge and then according to hydrophobicity (Washburn et al., 2001). Other types of resin, e.g., hydrophilic interaction liquid chromatography (HILIC) and size-exclusion chromatography (SEC) have also been used in proteomic studies (Gilar et al., 2005a). More recently, RP–RP HPLC systems using high pH and low pH mobile phases in the first and second separation dimensions, respectively, have proved to be excellent and robust for proteomics work (Gilar et al., 2005b). This set up can be fully automated and is suitable for proteomics work where several biological replicates are needed. It can be combined with both label based and label-free quantification methods. It is also possible to achieve absolute quantification of the identified proteins by spiking in known amounts of digested protein standards (Silva et al., 2006). Separation using only one dimension is also possible, but for complex samples or samples with high dynamic range, the number of protein identifications will be limited due to lower peak capacity compared to 2D LC strategies where two orthogonally retention mechanisms are used.

Mass spectrometry data contains peptide information at the MS and at the MS/MS level. For protein identification the MS and MS/MS data can be searched using commercial or publicly available search engines such as Sequest, Mascot, OMSSA, or X!tandem (Cottrell, 2011). Software designed for handling large proteomics datasets integrates multiple features such as identification, quantification, visualization, statistics, and reporting. These include packages such as Phenyx, Trans-Proteomic Pipeline (TPP) MaxQuant, and Peaks (Lemeer et al., 2012).

## CURRENT STATUS OF BARLEY PROTEOMICS

The areas where barley proteomics has been used can be divided into (a) industry driven biotechnology, including seed germination and maturation, beer proteomes, and malting proteomes and (b) biology driven proteomics covering plant adaptation to abiotic stress and organelle function including the chloroplast that is the focus of this review.

*Biotechnology driven proteomics*: Understanding the mechanisms involved in seed germination and maturation processes are important aspects in the malting industry where, e.g., enzyme amount such as amylase in different cultivars influences the conversion of starch into fermentable sugars. The work with proteome analysis of different barley seed cultivars and proteomes from different developmental stages of germinating barley started in year 2002 (Finnie et al., 2002; Ostergaard et al., 2002). 2D gels were used as a protein profiling tool. The proteins were extracted using a low salt buffer, favoring the extraction of water soluble seed proteins such as amylases and chitinases, and minimized extraction of high abundant storage proteins such as hordeins that otherwise would dominate the protein profile in the 2D gel. The TrEMBL database at that time only contained 546 barley protein sequences, so therefore most of the protein identifications were based on cross-species protein annotation using other cereals, such as rice, maize, and wheat. The strength of 2D gel electrophoresis was also pointed out in these studies, since the same protein was identified in multiple protein spots, maybe as a consequence of post-translational modifications (PTM) or multiple alleles with almost identical protein sequences.

Hynek et al. (2009) reported the enrichment of hydrophobic membrane proteins from the barley plasma membrane fraction, which may play a key role in the germination process, by using two-phase partitioning and RP chromatography. The enrichment of the membrane fraction was validated using western blotting against H^+^-ATPase, a protein located in the membrane. Sixty-one barley proteins were identified after SDS-PAGE by using electrospray tandem mass spectrometry (ESI-MS/MS).

Protein profiles of different beers are diverse due to differences in the barley cultivar, the malting process and the brewing yeast. 2D gel maps of different beer proteomes representing different cultivars and malting types have been created. The maps can be used as quality control step in the brewing industry and as a tool to detect and identify beer type specific proteins or protein isoforms that might represent taste, flavor, or texture. In the long term this will potentially enable manipulation of, e.g., flavor proteins (Fasoli et al., 2010; Iimure et al., 2010). The industrial induced protein modification called Maillard reactions has also been monitored and characterized and is important for color, taste, and flavor and include thermal stability of proteins and the non-enzymatic glycation of proteins (Perrocheau et al., 2005; Okada et al., 2008; Petry-Podgorska et al., 2010)**

*Biology driven proteomics*: 2D gel electrophoresis was the preferred method to study the proteome of barley plants exposed to salinity stress and adaptation (Fatehi et al., 2012). Barley plants, a tolerant and a salt-sensitive genotype, were exposed to 0 (control) or 300 mM NaCl. More than 500 reproducible protein spots were detected of which 44 appeared to be regulated. The regulated proteins were involved in several biological processes such as reactive oxygen species scavenging, signal transduction, and protein processing. The advantage of this 2D gel strategy for studying a non-sequenced organism was pointed out – only the regulated proteins needed to be analyzed and identified by mass spectrometry. A similar procedure was used in a nitrogen use efficiency study of barley, where proteomes from barley shoots and roots were analyzed using 2D gels. Comparative proteome analysis of plants grown with a nitrogen source and plants grown under nitrogen deficiency revealed 67 and 49 differentially regulated protein spots in roots and shoots, respectively (Moller et al., 2011a). Proteins associated with drought have also been analyzed using 2D gel proteomics (Wendelboe-Nelson and Morris, 2012). In a comparative study of barley, extracted leave and root proteomes from boron tolerant and boron intolerant barley plants were studied using an iTRAQ based method and peptide fractionation by 2D LC prior to mass spectrometry analysis. A total of 138 proteins were identified from leaf tissue and 341 were identified from root tissues. Only 11 out of 1038 peptides from the root tissue were regulated in the boron tolerant barley plant. Interestingly seven of these peptides identified three proteins involved in iron deficiency response (Patterson et al., 2007).

Protein modifications such as acetylation, glycosylation, and phosphorylation are important regulators of a wide range of biological processes in plants (Ytterberg and Jensen, 2010). In barley only a handful of proteomics studies deal with protein modifications. These include protein characterization in seeds during maturation using 2D gels (Finnie et al., 2006; Laugesen et al., 2007), where spot “trains” of the same proteins appeared during maturation as a consequence of small amino acids sequence differences, processing and differences in the degree of protein glycosylation. Phosphoprotein studies in tonoplasts revealed a total of 65 phosphopeptides, and provide a first view into the regulation of several metabolic pathways in tonoplast (Endler et al., 2009). Phosphoproteomics in plants were recently reviewed (Kline-Jonakin et al., 2011).

### THE BARLEY CHLOROPLAST PROTEOME

**FIGURE 2:**
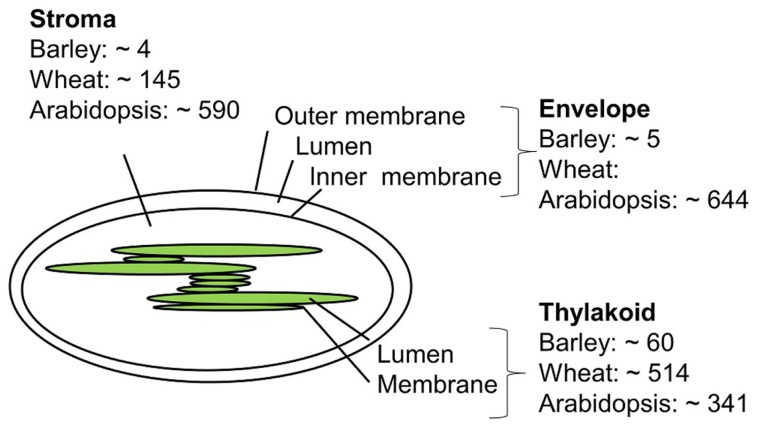
**Approximate number of chloroplast proteins identified in barley (Ciambella et al., 2005; Bartsch et al., 2008; Ploscher et al., 2011), wheat (Kamal et al., 2012), and Arabidopsis (Peltier et al., 2004; Giacomelli et al., 2006; Zybailov et al., 2008; Ferro et al., 2010)**.

Only a few studies concerning the barley chloroplast proteome have been published, and a comprehensive list of barley chloroplast proteins is yet to be reported. In contrast, global proteomics in *Arabidopsis* has been a reality for more than 20 years due to the complete sequencing of the *A. thaliana* genome at the beginning of this millennium. (Kaul et al., 2000; Wortman et al., 2003). Chloroplast proteome work in barley, wheat, and *A. thaliana* will be discussed below. **Figure 2** compares the number of proteins identified in the chloroplast sub-compartments from these three species.

*The envelope membrane: *The envelope membrane of the chloroplast is the site of several important functions such as biosynthesis of glycerolipids, fatty acid export, metabolite transport, and protein import. In *A. thaliana*, Ferro et al. (2010) reported 644 proteins to be associated with the membrane envelope using both in-gel and in-solution digestion of proteins. Earlier studies of the envelope membrane using both geLC-MS/MS and 2D LC-MS/MS produced fewer identifications (Ferro et al., 2003; Froehlich et al., 2003).

*The thylakoid membrane:* The thylakoid membrane contains the photosynthetic machinery, but also proteins involved in regulation and maintenance of this machinery. In thylakoid preparations from *A. thaliana* the number of identified proteins sums up to 242 using geLC-MS/MS and LC-MS/MS and 154 proteins using 2D gels (Friso et al., 2004; Peltier et al., 2004). A total of 198 thylakoid luminal proteins have been identified combing data from several studies (Peltier et al., 2002; Giacomelli et al., 2006). Some of these are believed to be up to 10,000-fold less abundant than photosynthetic proteins, and can only be identified by sub-proteome isolation. Other studies on luminal proteins report less proteins (Schubert et al., 2002), which might reflect differences in purification and proteomics strategies.

The thylakoid membrane of barley was investigated by the use of BN 2D PAGE, with the aim to compare the photosynthetic machinery of barley with that of other higher plants (Ciambella et al., 2005). The number of barley thylakoid proteins identified was 45, of these 17 proteins from photosystem II (PSII), 16 from PSI, 7 proteins from cytochrome B6, and 5 from the ATP synthase. The same number of barley thylakoid proteins was reached in another study (Granvogl et al., 2006). One recent study from 2011 (Ploscher et al., 2011) compares protein complexes from etioplast and chloroplast. This is at the moment the most comprehensive chloroplast proteome study in barley. Etioplasts develop in the absence of light but can mature into chloroplasts by illumination. By using 2D BN/SDS-PAGE to separate the protein membrane complexes from etioplast and chloroplast, they found eight etioplast/chloroplast shared protein complexes, among those with high number of subunit representation were the ATPase, cytochrome b6, and the NAD(P)H dehydrogenase complex, whereas the PSI and PSII complexes were only present in the chloroplast. The use of BN gels made it possible to quantify and distinguish between monomeric, dimeric, and multimeric forms of the photosynthetic protein machinery, and to distinguish between the different subunits present in the protein complexes, making assumptions of assembly and maturation of protein complexes possible. Both automated and manually inspected fragment spectra were generated from the mass spectrometry based analysis where both online protein identification of tryptic digested proteins and off-line identification of intact small proteins extraction from gel were identified. In an earlier study by the same group (Ploscher et al., 2009), intact low molecular weight proteins from PSII were identified using off-line ESI MS.

*The stroma:* The stroma contains the genetic material and important metabolic enzymes including those involved in the Calvin cycle. Using the geLC-MS/MS approach a total of 590 *A. thaliana* proteins were identified (Zybailov et al., 2008). Less protein identifications were obtained in an attempt to identify paralogs using 2D native gels (Peltier et al., 2006). For barley no stromal proteome studies have appeared to date, but four proteins from the above mentioned preparations (Ciambella et al., 2005; Ploscher et al., 2011) are supposedly targeted to the stroma.

In a recent chloroplast proteomic study in wheat, which shares sequence similarity with barley, the geLC-MS/MS strategy was used, and a total of 607 chloroplast proteins were identified. Of these, 145 were from stroma, 342 were from the thylakoid membrane, 163 from the lumen, and 166 proteins were integral membrane proteins (Kamal et al., 2012).

Armbruster et al. (2011) summarizes all proteomics work on chloroplast and comes up with a total number of nucleus encoded proteins to be 1741, 63% with predicted cTP.

## THE FUTURE FOR PROTEOMICS OF BARLEY AND BARLEY CHLOROPLASTS

Proteomics work in barley has to date been hampered by the lack of complete genomic sequence. But by the complete sequencing of the barley genome the goal to identify all of the predicted 2000–3000 chloroplast protein is within reach. The shift in analytical methods in proteomics from 2D gels toward 2D LC-MS/MS based strategies, due to completely sequenced genomes, improved nano-LC systems and faster and more sensitive tandem mass spectrometers has over the years increased the output of proteomics data. We foresee that new robust in-solution digestion protocol coupled with fast online 2D LC-MS/MS systems will enable the next major step in barley proteomics by decreasing workload and increasing the throughput, identification rate and accuracy of quantitation of the proteomics technologies.

In the near future we expect to see more quantitative proteomics studies of barley, e.g., for molecular analysis of abiotic stress, where sensitive versus non-sensitive barley genotypes are compared, with the aim of identifying protein biomarker involved in a certain genotypic trait. Ultimately this would couple proteomics and other technologies into the multidisciplinary systems biology platform in the pursuit of sustainable crop production.

## Conflict of Interest Statement

The authors declare that the research was conducted in the absence of any commercial or financial relationships that could be construed as a potential conflict of interest.

## References

[B1] Abdalla K. O. Rafudeen M. S. (2012). Analysis of the nuclear proteome of the resurrection plant *Xerophyta viscosa* in response to dehydration stress using iTRAQ with 2DLC and tandem mass spectrometry. *J. Proteomics* 75 2361–2374 22361341 10.1016/j.jprot.2012.02.006

[B2] Aebersold R. Mann M. (2003). Mass spectrometry-based proteomics. *Nature* 422 198–207 12634793 10.1038/nature01511

[B3] Agrawal G. K. Bourguignon J. Rolland N. Ephritikhine G. Ferro M. Jaquinod M. (2011). Plant organelle proteomics: collaborating for optimal cell function. *Mass Spectrom. Rev.* 30 772–853 10.1002/mas.20301 21038434

[B4] Armbruster U. Pesaresi P. Pribil M. Hertle A. Leister D. (2011). Update on chloroplast research: new tools, new topics, and new trends. *Mol. Plant* 4 1–16 20924030 10.1093/mp/ssq060

[B5] Aronsson H. Jarvis P. (2002). A simple method for isolating import-competent *Arabidopsis* chloroplasts. *FEBS Lett.* 529 215–220 12372603 10.1016/s0014-5793(02)03342-2

[B6] Ayoubi T. A. Y Van De Ven W. J. M. (1996). Regulation of gene expression by alternative promoters. *FASEB J.* 10 453–460 8647344

[B7] Bannai H. Tamada Y. Maruyama O. Nakai K. Miyano S. (2002). Extensive feature detection of N-terminal protein sorting signals. *Bioinformatics* 18 298–305 11847077 10.1093/bioinformatics/18.2.298

[B8] Bantscheff M. Lemeer S. Savitski M. M. Kuster B. (2012). Quantitative mass spectrometry in proteomics: critical review update from 2007 to the present. *Anal. Bioanal. Chem.* 404 939–965 22772140 10.1007/s00216-012-6203-4

[B9] Bantscheff M. Schirle M. Sweetman G. Rick J. Kuster B. (2007). Quantitative mass spectrometry in proteomics: a critical review. *Anal. Bioanal. Chem.* 389 1017–1031 17668192 10.1007/s00216-007-1486-6

[B10] Bartsch S. Monnet J. Selbach K. Quigley F. Gray J. von Wettstein D. (2008). Three thioredoxin targets in the inner envelope membrane of chloroplasts function in protein import and chlorophyll metabolism. *Proc. Natl. Acad. Sci. U.S.A.* 105 4933–4938 18349143 10.1073/pnas.0800378105 PMC2290756

[B11] Bensimon A. Heck A. J. Aebersold R. (2012). Mass spectrometry-based proteomics and network biology. *Annu. Rev. Biochem.* 81 379–405 22439968 10.1146/annurev-biochem-072909-100424

[B12] Berth M. Moser F. M. Kolbe M. Bernhardt J. (2007). The state of the art in the analysis of two-dimensional gel electrophoresis images. *Appl. Microbiol. Biotechnol.* 76 1223–1243 17713763 10.1007/s00253-007-1128-0 PMC2279157

[B13] Bindschedler L. V. Cramer R. (2011). Quantitative plant proteomics. *Proteomics* 11 756–775 21246733 10.1002/pmic.201000426

[B14] Bindschedler L. V. Palmblad M. Cramer R. (2008). Hydroponic isotope labelling of entire plants (HILEP) for quantitative plant proteomics; an oxidative stress case study. *Phytochemistry* 69 1962–1972 18538804 10.1016/j.phytochem.2008.04.007

[B15] Boden M. Hawkins J. (2005). Prediction of subcellular localization using sequence-biased recurrent networks. *Bioinformatics* 21 2279–2286 15746276 10.1093/bioinformatics/bti372

[B16] Boschetti E. Righetti P. G. (2008). The ProteoMiner in the proteomic arena: a non-depleting tool for discovering low-abundance species. *J. Proteomics* 71 255–264 18603494 10.1016/j.jprot.2008.05.002

[B17] Cellar N. A. Kuppannan K. Langhorst M. L. Ni W. Xu P. Young S. A. (2008). Cross species applicability of abundant protein depletion columns for ribulose-1,5-bisphosphate carboxylase/oxygenase. *J. Chromatogr. B Analyt. Technol. Biomed. Life Sci.* 861 29–39 10.1016/j.jchromb.2007.11.024 18063427

[B18] Chen M. J. Mooney B. P. Hajduch M. Joshi T. Zhou M. Y. Xu D. (2009). System analysis of an *Arabidopsis* mutant altered in de novo fatty acid synthesis reveals diverse changes in seed composition and metabolism. *Plant Physiol.* 150 27–41 19279196 10.1104/pp.108.134882 PMC2675738

[B19] Chevalier F. (2010). Highlights on the capacities of “Gel-based” proteomics. *Proteome Sci.* 8 23 10.1186/1477-5956-8-23 PMC2873371 20426826

[B20] Ciambella C. Roepstorff P. Aro E. M. Zolla L. (2005). A proteomic approach for investigation of photosynthetic apparatus in plants. *Proteomics* 5 746–757 15682463 10.1002/pmic.200401129

[B21] Cottrell J. S. (2011). Protein identification using MS/MS data. *J. Proteomics* 74 1842–1851 21635977 10.1016/j.jprot.2011.05.014

[B22] Cravatt B. F. Simon G. M Yates J. R. III (2007). The biological impact of mass-spectrometry-based proteomics. *Nature* 450 991–1000 18075578 10.1038/nature06525

[B23] de Hoog C. L. Mann M. (2004). Proteomics. *Annu. Rev. Genomics Hum. Genet.* 5 267–293 15485350 10.1146/annurev.genom.4.070802.110305

[B24] Dreher K. Callis J. (2007). Ubiquitin, hormones and biotic stress in plants. *Ann. Bot.* 99 787–822 17220175 10.1093/aob/mcl255 PMC2802907

[B25] Emanuelsson O. Nielsen H. Von Heijne G. (1999). ChloroP, a neural network-based method for predicting chloroplast transit peptides and their cleavage sites. *Protein Sci.* 8 978–984 10338008 10.1110/ps.8.5.978 PMC2144330

[B26] Endler A. Reiland S. Gerrits B. Schmidt U. G. Baginsky S. Martinoia E. (2009). In vivo phosphorylation sites of barley tonoplast proteins identified by a phosphoproteomic approach. *Proteomics* 9 310–321 19142958 10.1002/pmic.200800323

[B27] Eriksson J. Fenyo D. (2010). “Modeling experimental design for proteomics,” in *Computational Biology* ed Fenyo D. (Totowa: Humana Press Inc.) 223–230 10.1007/978-1-60761-842-3_14 PMC3745767 20835802

[B28] Fasoli E. Aldini G. Regazzoni L. Kravchuk A. V. Citterio A. Righetti P. G. (2010). Les maitres de l’Orge: the proteome content of your beer mug. *J. Proteome Res.* 9 5262–5269 20722451 10.1021/pr100551n

[B29] Fatehi F. Hosseinzadeh A. Alizadeh H. Brimavandi T. Struik P. C. (2012). The proteome response of salt-resistant and salt-sensitive barley genotypes to long-term salinity stress. *Mol. Biol. Rep.* 39 6387–6397 22297690 10.1007/s11033-012-1460-z

[B30] Ferro M. Brugiere S. Salvi D. Seigneurin-Berny D. Court M. Moyet L. (2010). AT_CHLORO, a comprehensive chloroplast proteome database with subplastidial localization and curated information on envelope proteins. *Mol. Cell. Proteomics* 9 1063–1084 20061580 10.1074/mcp.M900325-MCP200 PMC2877971

[B31] Ferro M. Salvi D. Brugiere S. Miras S. Kowalski S. Louwagie M. (2003). Proteomics of the chloroplast envelope membranes from *Arabidopsis thaliana*. *Mol. Cell. Proteomics* 2 325–345 12766230 10.1074/mcp.M300030-MCP200

[B32] Fido R. J. Mills E. N. Rigby N. M. Shewry P. R. (2004). Protein extraction from plant tissues. *Methods Mol. Biol.* 244 21–27 14970539 10.1385/1-59259-655-x:21

[B33] Finnie C. Bak-Jensen K. S. Laugesen S. Roepstorff P. Svensson B. (2006). Differential appearance of isoforms and cultivar variation in protein temporal profiles revealed in the maturing barley grain proteome. *Plant Sci.* 170 808–821

[B34] Finnie C. Melchior S. Roepstorff P. Svensson B. (2002). Proteome analysis of grain filling and seed maturation in barley. *Plant Physiol.* 129 1308–1319 12114584 10.1104/pp.003681 PMC166524

[B35] Friso G. Giacomelli L. Ytterberg A. J. Peltier J. B. Rudella A. Sun Q. (2004). In-depth analysis of the thylakoid membrane proteome of *Arabidopsis thaliana* chloroplasts: new proteins, new functions, and a plastid proteome database. *Plant Cell* 16 478–499 14729914 10.1105/tpc.017814 PMC341918

[B36] Froehlich J. E. Wilkerson C. G. Ray W. K. McAndrew R. S. Osteryoung K. W. Gage D. A. (2003). Proteomic study of the *Arabidopsis thaliana* chloroplastic envelope membrane utilizing alternatives to traditional two-dimensional electrophoresis. *J. Proteome Res.* 2 413–425 12938931 10.1021/pr034025j

[B37] Frohlich A. Gaupels F. Sarioglu H. Holzmeister C. Spannagl M. Durner J. (2012). Looking deep inside: detection of low-abundance proteins in leaf extracts of *Arabidopsis* and phloem exudates of pumpkin. *Plant Physiol.* 159 902–914 22555880 10.1104/pp.112.198077 PMC3387715

[B38] Gerber S. A. Rush J. Stemman O. Kirschner M. W. Gygi S. P. (2003). Absolute quantification of proteins and phosphoproteins from cell lysates by tandem MS. *Proc. Natl. Acad. Sci. U.S.A.* 100 6940–6945 12771378 10.1073/pnas.0832254100 PMC165809

[B39] Gevaert K. Vandekerckhove J. (2000). Protein identification methods in proteomics. *Electrophoresis* 21 1145–1154 10786887 10.1002/(SICI)1522-2683(20000401)21:6<1145::AID-ELPS1145>3.0.CO;2-Z

[B40] Giacomelli L. Rudella A. van Wijk K. J. (2006). High light response of the thylakoid proteome in *Arabidopsis* wild type and the ascorbate-deficient mutant vtc2-2. A comparative proteomics study. *Plant Physiol.* 141 685–701 10.1104/pp.106.080150 PMC1475442 16648217

[B41] Gilar M. Olivova P. Daly A. E. Gebler J. C. (2005a). Orthogonality of separation in two-dimensional liquid chromatography. *Anal. Chem.* 77 6426–6434 16194109 10.1021/ac050923i

[B42] Gilar M. Olivova P. Daly A. E. Gebler J. C. (2005b). Two-dimensional separation of peptides using RP-RP-HPLC system with different pH in first and second separation dimensions. *J. Sep. Sci.* 28 1694–1703 16224963 10.1002/jssc.200500116

[B43] Goff S. A. Ricke D. Lan T. H. Presting G. Wang R. L. Dunn M. (2002). A draft sequence of the rice genome (*Oryza sativa* L. ssp japonica). *Science* 296 92–100 11935018 10.1126/science.1068275

[B44] Gouw J. W. Tops B. B. Mortensen P. Heck A. J. Krijgsveld J. (2008). Optimizing identification and quantitation of 15N-labeled proteins in comparative proteomics. *Anal. Chem.* 80 7796–7803 18808151 10.1021/ac801249v

[B45] Granvogl B. Reisinger V. Eichacker L. A. (2006). Mapping the proteome of thylakoid membranes by de novo sequencing of intermembrane peptide domains. *Proteomics* 6 3681–3695 16758444 10.1002/pmic.200500924

[B46] Gruhler A. Schulze W. X. Matthiesen R. Mann M. Jensen O. N. (2005). Stable isotope labeling of *Arabidopsis thaliana* cells and quantitative proteomics by mass spectrometry. *Mol. Cell. Proteomics* 4 1697–1709 16088002 10.1074/mcp.M500190-MCP200

[B47] Hall M. Mishra Y. Schröder W. P. (2011). “Preparation of stroma, thylakoid membrane, and lumen fractions from *Arabidopsis thaliana* chloroplasts for proteomic analysis.” in *Chloroplast Research in Arabidopsis* ed. Paul Jarvis R. (New York: Humana Press) 207–222 10.1007/978-1-61779-237-3_11 21863445

[B48] Horton P. Park K. J. Obayashi T. Fujita N. Harada H. Adams-Collier C. J. (2007). WoLF PSORT: protein localization predictor. *Nucleic Acids Res.* 35 W585–W587 17517783 10.1093/nar/gkm259 PMC1933216

[B49] Hynek R. Svensson B. Jensen O. N. Barkholt V. Finnie C. (2009). The plasma membrane proteome of germinating barley embryos. *Proteomics* 9 3787–3794 19639596 10.1002/pmic.200800745

[B50] Iimure T. Nankaku N. Hirota N. Zhou T. S. Hoki T. Kihara M. (2010). Construction of a novel beer proteome map and its use in beer quality control. *Food Chem.* 118 566–574

[B51] Issaq H. Veenstra T. (2008). Two-dimensional polyacrylamide gel electrophoresis (2D-PAGE): advances and perspectives. *Biotechniques* 44 697–698, 700. 18474047 10.2144/000112823

[B52] Jacob A. M. Turck C. W. (2008). Detection of post-translational modifications by fluorescent staining of two-dimensional gels. *Methods Mol. Biol.* 446 21–32 18373247 10.1007/978-1-60327-084-7_2

[B53] James P. (1997). Protein identification in the post-genome era: the rapid rise of proteomics. *Q. Rev. Biophys.* 30 279–331 9634650 10.1017/s0033583597003399

[B54] Jones A. M. E. Bennett M. H. Mansfield J. W. Grant M. (2006). Analysis of the defence phosphoproteome of *Arabidopsis thaliana* using differential mass tagging. *Proteomics* 6 4155–4165 16850419 10.1002/pmic.200500172

[B55] Jorrin J. V. Maldonado A. M. Castillejo M. A. (2007). Plant proteome analysis: a 2006 update. *Proteomics* 7 2947–2962 17654459 10.1002/pmic.200700135

[B56] Kamal A. M. Cho K. Komatsu S. Uozumi N. Choi J. S. Woo S. H. (2012). Towards an understanding of wheat chloroplasts: a methodical investigation of thylakoid proteome. *Mol. Biol. Rep.* 39 5069–5083 22160430 10.1007/s11033-011-1302-4

[B57] Kaul S. Koo H. L. Jenkins J. Rizzo M. Rooney T. Tallon L. J. (2000). Analysis of the genome sequence of the flowering plant *Arabidopsis thaliana*. *Nature* 408 796–815 11130711 10.1038/35048692

[B58] Kieselbach T. Bystedt M. Hynds P. Robinson C. Schroder W. P. (2000). A peroxidase homologue and novel plastocyanin located by proteomics to the *Arabidopsis* chloroplast thylakoid lumen. *FEBS Lett.* 480 271–276 11034343 10.1016/s0014-5793(00)01890-1

[B59] Kieselbach T. Hagman Andersson B. Schroder W. P. (1998). The thylakoid lumen of chloroplasts. Isolation and characterization. *J. Biol. Chem.* 273 6710–6716 9506969 10.1074/jbc.273.12.6710

[B60] Kleffmann T. Russenberger D. von Zychlinski A. Christopher W. Sjolander K. Gruissem W. (2004). The *Arabidopsis thaliana* chloroplast proteome reveals pathway abundance and novel protein functions. *Curr. Biol.* 14 354–362 15028209 10.1016/j.cub.2004.02.039

[B61] Kline-Jonakin K. G. Barrett-Wilt G. A. Sussman M. R. (2011). Quantitative plant phosphoproteomics. *Curr. Opin. Plant Biol.* 14 507–511 21764629 10.1016/j.pbi.2011.06.008 PMC3253357

[B62] Krause F. (2006). Detection and analysis of protein-protein interactions in organellar and prokaryotic proteomes by native gel electrophoresis: (Membrane) protein complexes and supercomplexes. *Electrophoresis* 27 2759–2781 16817166 10.1002/elps.200600049

[B63] Krishnan H. B. Natarajan S. S. (2009). A rapid method for depletion of Rubisco from soybean (*Glycine max*) leaf for proteomic analysis of lower abundance proteins. *Phytochemistry* 70 1958–1964 19766275 10.1016/j.phytochem.2009.08.020

[B64] Kuntz M. Rolland N. (2012). “Subcellular and sub-organellar proteomics as a complementary tool to study the evolution of the plastid proteome,” *Organelle Genetics: Evolution of Organelle Genomes and Gene Expression* ed Bullerwell C. E. (New York: Springer).

[B65] Lander E. S. Linton L. M. Birren B. Nusbaum C. Zody M. C. Baldwin J. (2001). Initial sequencing and analysis of the human genome. *Nature* 409 860–921 11237011 10.1038/35057062

[B66] Larsen M. R. Trelle M. B. Thingholm T. E. Jensen O. N. (2006). Analysis of posttranslational modifications of proteins by tandem mass spectrometry. *Biotechniques* 40 790–798 16774123 10.2144/000112201

[B67] Laugesen S. Bak-Jensen K. S. Hagglund P. Henriksen A. Finnie C. Svensson B. (2007). Barley peroxidase isozymes – expression and post-translational modification in mature seeds as identified by two-dimensional gel electrophoresis and mass spectrometry. *Int. J. Mass Spectrom.* 268 244–253

[B68] Lemeer S. Hahne H. Pachl F. Kuster B. (2012). Software tools for MS-based quantitative proteomics: a brief overview. *Methods Mol. Biol.* 893 489–499 22665318 10.1007/978-1-61779-885-6_29

[B69] Lin Y. Liu H. Liu Z. H. Wang X. C. Liang S. P. (2012). Shotgun analysis of membrane proteomes using a novel combinative strategy of solution-based sample preparation coupled with liquid chromatography-tandem mass spectrometry. *J. Chromatogr. B Analyt. Technol. Biomed. Life Sci.* 901 18–24 10.1016/j.jchromb.2012.05.035 22721709

[B70] Mano S. Miwa T. Nishikawa S. Mimura T. Nishimura M. (2008). The plant organelles database (PODB): a collection of visualized plant organelles and protocols for plant organelle research. *Nucleic Acids Res.* 36 929–937 17932059 10.1093/nar/gkm789 PMC2238956

[B71] Manza L. L. Stamer S. L. Ham A. J. L. Codreanu S. G. Liebler D. C. (2005). Sample preparation and digestion for proteomic analyses using spin filters. *Proteomics* 5 1742–1745 15761957 10.1002/pmic.200401063

[B72] Mayer K. F. Waugh R. Langridge P. Close T. J. Wise R. P. Graner A. (2012). A physical, genetic and functional sequence assembly of the barley genome. *Nature*. 491 711–716 23075845 10.1038/nature11543

[B73] Melo-Braga M. N. Verano-Braga T. Leon I. R. Antonacci D. Nogueira F. C. S. Thelen J. J. (2012). Modulation of protein phosphorylation, *N*-glycosylation and Lys-acetylation in grape (*Vitis vinifera*) mesocarp and exocarp owing to *Lobesia botrana* infection. *Mol. Cell. Proteomics* 11 945–956 22778145 10.1074/mcp.M112.020214 PMC3494143

[B74] Metodiev M. Demirevskakepova K. (1992). Rbisco quantitation in leaves of different barley varieties by enzyme-linked-immunosorbent-assay. *J. Exp. Bot.* 43 155–158

[B75] Millar A. H. Heazlewood J. L. Kristensen B. K. Braun H. P. Moller I. M. (2005). The plant mitochondrial proteome. *Trends Plant Sci.* 10 36–43 15642522 10.1016/j.tplants.2004.12.002

[B76] Mithoe S. C Menke F. L. H. (2011). Phosphoproteomics perspective on plant signal transduction and tyrosine phosphorylation. *Phytochemistry* 72 997–1006 21315387 10.1016/j.phytochem.2010.12.009

[B77] Moller A. L. B. Pedas P. Andersen B. Svensson B. Schjoerring J. K. Finnie C. (2011a). Responses of barley root and shoot proteomes to long-term nitrogen deficiency, short-term nitrogen starvation and ammonium. *Plant Cell Environ.* 34 2024–2037 21736591 10.1111/j.1365-3040.2011.02396.x

[B78] Moller I. M. Rogowska-Wrzesinska A Rao R. S. P. (2011b). Protein carbonylation and metal-catalyzed protein oxidation in a cellular perspective. *J. Proteomics* 74 2228–2242 21601020 10.1016/j.jprot.2011.05.004

[B79] Nawrocki A. Thorup-Kristensen K. Jensen O. N. (2011). Quantitative proteomics by 2DE and MALDI MS/MS uncover the effects of organic and conventional cropping methods on vegetable products. *J. Proteomics* 74 2810–2825 21757040 10.1016/j.jprot.2011.06.021

[B80] Neilson K. A. Mariani M. Haynes P. A. (2011). Quantitative proteomic analysis of cold-responsive proteins in rice. *Proteomics* 11 1696–1706 21433000 10.1002/pmic.201000727

[B81] Nelson C. J. Huttlin E. L. Hegeman A. D. Harms A. C. Sussman M. R. (2007). Implications of N-15-metabolic labeling for automated peptide identification in *Arabidopsis* thaliana. *Proteomics* 7 1279–1292 17443642 10.1002/pmic.200600832

[B82] Neuburger M. Journet E. P. Bligny R. Carde J. P. Douce R. (1982). Purification of plant-mitochondria by isopycnic centrifugation in density gradients of percollLL. *Arch. Biochem. Biophys.* 217 312–323 6289753 10.1016/0003-9861(82)90507-0

[B83] Ng D. W. K. Zhang C. Miller M. Shen Z. Briggs S. P. Chen Z. J. (2012). Proteomic divergence in *Arabidopsis* autopolyploids and allopolyploids and their progenitors. *Heredity (Edinb)* 108 419–430 22009271 10.1038/hdy.2011.92 PMC3313054

[B84] Norrgran J. Williams T. L. Woolfitt A. R. Solano M. I. Pirkle J. L. Barr J. R. (2009). Optimization of digestion parameters for protein quantification. *Anal. Biochem.* 393 48–55 19501563 10.1016/j.ab.2009.05.050

[B85] Okada Y. Limure T. Takoi K. Kaneko T. Kihara M. Hayashi K. (2008). The influence of barley malt protein modification on beer foam stability and their relationship to the barley dimeric alpha-amylase inhibitor-1 (BDAI-1) as a possible foam-promoting protein. *J. Agric. Food Chem.* 56 1458–1464 18237135 10.1021/jf0724926

[B86] Ong S. E. Blagoev B. Kratchmarova I. Kristensen D. B. Steen H. Pandey A. (2002). Stable isotope labeling by amino acids in cell culture, SILAC, as a simple and accurate approach to expression proteomics. *Mol. Cell. Proteomics* 1 376–386 12118079 10.1074/mcp.m200025-mcp200

[B87] Ong S. E. Mann M. (2005). Mass spectrometry-based proteomics turns quantitative. *Nat. Chem. Biol.* 1 252–262 16408053 10.1038/nchembio736

[B88] Ostergaard O. Melchior S. Roepstorff P. Svensson B. (2002). Initial proteome analysis of mature barley seeds and malt. *Proteomics* 2 733–739 12112856 10.1002/1615-9861(200206)2:6<733::AID-PROT733>3.0.CO;2-E

[B89] Patterson J. Ford K. Cassin A. Natera S. Bacic A. (2007). Increased abundance of proteins involved in phytosiderophore production in boron-tolerant barley. *Plant Physiol.* 144 1612–1631 17478636 10.1104/pp.107.096388 PMC1914127

[B90] Patterson S. D. Aebersold R. H. (2003). Proteomics: the first decade and beyond. *Nat. Genet.* 33 311–323 12610541 10.1038/ng1106

[B91] Patton W. F. (2002). Detection technologies in proteome analysis. *J. Chromatogr B Analyt. Technol. Biomed. Life Sci.* 771 3–31 10.1016/s1570-0232(02)00043-0 12015990

[B92] Peltier J. B. Cai Y. Sun Q. Zabrouskov V. Giacomelli L. Rudella A. (2006). The oligomeric stromal proteome of *Arabidopsis thaliana* chloroplasts. *Mol. Cell. Proteomics* 5 114–133 16207701 10.1074/mcp.M500180-MCP200

[B93] Peltier J. B. Emanuelsson O. Kalume D. E. Ytterberg J. Friso G. Rudella A. (2002). Central functions of the lumenal and peripheral thylakoid proteome of *Arabidopsis* determined by experimentation and genome-wide prediction. *Plant Cell* 14 211–236 11826309 10.1105/tpc.010304 PMC150561

[B94] Peltier J. B. Friso G. Kalume D. E. Roepstorff P. Nilsson F. Adamska I. (2000). Proteomics of the chloroplast: systematic identification and targeting analysis of lumenal and peripheral thylakoid proteins. *Plant Cell* 12 319–341 10715320 10.1105/tpc.12.3.319 PMC139834

[B95] Peltier J. B. Ytterberg A. J. Sun Q. van Wijk K. J. (2004). New functions of the thylakoid membrane proteome of *Arabidopsis thaliana* revealed by a simple, fast, and versatile fractionation strategy. *J. Biol. Chem.* 279 49367–49383 15322131 10.1074/jbc.M406763200

[B96] Perrocheau L. Rogniaux H. Boivin P. Marion D. (2005). Probing heat-stable water-soluble proteins from barley to malt and beer. *Proteomics* 5 2849–2858 15986330 10.1002/pmic.200401153

[B97] Petry-Podgorska I. Zidkova J. Flodrova D. Bobalova J. (2010). 2D-HPLC and MALDI-TOF/TOF analysis of barley proteins glycated during brewing. *J. Chromatogr. B Analyt. Technol. Biomed. Life Sci.* 878 3143–3148 10.1016/j.jchromb.2010.09.023 20956095

[B98] Ploscher M. Granvogl B. Zoryan M. Reisinger V. Eichacker L. A. (2009). Mass spectrometric characterization of membrane integral low molecular weight proteins from photosystem II in barley etioplasts. *Proteomics* 9 625–635 19137553 10.1002/pmic.200800337

[B99] Ploscher M. Reisinger V. Eichacker L. A. (2011). Proteomic comparison of etioplast and chloroplast protein complexes. *J. Proteomics* 74 1256–1265 21440687 10.1016/j.jprot.2011.03.020

[B100] Podwojski K. Stephan C. Eisenacher M. (2012). Important issues in planning a proteomics experiment: statistical considerations of quantitative proteomic data. *Methods Mol. Biol.* 893 3–21 22665290 10.1007/978-1-61779-885-6_1

[B101] Pottiez G. Wiederin J. Fox H. S. Ciborowski P. (2012). Comparison of 4-plex to 8-plex iTRAQ quantitative measurements of proteins in human plasma samples. *J. Proteome Res.* 11 3774–3781 22594965 10.1021/pr300414z PMC3390908

[B102] Reisinger V. Eichacker L. A. (2007). How to analyze protein complexes by 2D blue native SDS-PAGE. *Proteomics* 7(Suppl. 1) 6–16 10.1002/pmic.200700205 17893852

[B103] Ross P. L. Huang Y. L. N. Marchese J. N. Williamson B. Parker K. Hattan S. (2004). Multiplexed protein quantitation in *Saccharomyces cerevisiae* using amine-reactive isobaric tagging reagents. *Mol. Cell. Proteomics* 3 1154–1169 15385600 10.1074/mcp.M400129-MCP200

[B104] Sachon E. Mohammed S. Bache N. Jensen O. N. (2006). Phosphopeptide quantitation using amine-reactive isobaric tagging reagents and tandem mass spectrometry: application to proteins isolated by gel electrophoresis. *Rapid Commun. Mass Spectrom.* 20 1127–1134 16521170 10.1002/rcm.2427

[B105] Saeed M. Dahab A. H. A. Guo W. Z. Zhang T. Z. (2012). A cascade of recently discovered molecular mechanisms involved in abiotic stress tolerance of plants. *OMICS* 16 188–199 22433075 10.1089/omi.2011.0109

[B106] Saisho D. Takeda K. (2011). Barley: emergence as a new research material of crop science. *Plant Cell Physiol.* 52 724–727 21565909 10.1093/pcp/pcr049

[B107] Schmidt A. Kellermann J. Lottspeich F. (2005). A novel strategy for quantitative proteomics using isotope-coded protein labels. *Proteomics* 5 4–15 15602776 10.1002/pmic.200400873

[B108] Schubert M. Petersson U. A. Haas B. J. Funk C. Schroder W. P. Kieselbach T. (2002). Proteome map of the chloroplast lumen of *Arabidopsis thaliana*. *J. Biol. Chem.* 277 8354–8365 11719511 10.1074/jbc.M108575200

[B109] Schulte D. Close T. J. Graner A. Langridge P. Matsumoto T. Muehlbauer G. (2009). The international barley sequencing consortium-at the threshold of efficient access to the barley genome. *Plant Physiol.* 149 142–147 19126706 10.1104/pp.108.128967 PMC2613708

[B110] Shevchenko A. Tomas H. Havlis J. Olsen J. V. Mann M. (2006). In-gel digestion for mass spectrometric characterization of proteins and proteomes. *Nat. Protoc.* 1 2856–2860 17406544 10.1038/nprot.2006.468

[B111] Shevchenko A. Wilm M. Vorm O. Mann M. (1996). Mass spectrometric sequencing of proteins from silver stained polyacrylamide gels. *Anal. Chem.* 68 850–858 8779443 10.1021/ac950914h

[B112] Silva J. C. Gorenstein M. V. Li G. Z. Vissers J. P. C. Geromanos S. J. (2006). Absolute quantification of proteins by LCMSE – a virtue of parallel MS acquisition. *Mol. Cell. Proteomics* 5 144–156 16219938 10.1074/mcp.M500230-MCP200

[B113] Small I. Peeters N. Legeai F. Lurin C. (2004). Predotar: a tool for rapidly screening proteomes for N-terminal targeting sequences. *Proteomics* 4 1581–1590 15174128 10.1002/pmic.200300776

[B114] Speers A. E. Wu C. C. (2007). Proteomics of integral membrane proteins theory and application. *Chem. Rev.* 107 3687–3714 17683161 10.1021/cr068286z

[B115] Syed N. H. Kalyna M. Marquez Y. Barta A. Brown J. W. S. (2012). Alternative splicing in plants – coming of age. *Trends Plant Sci.* 17 616–623 22743067 10.1016/j.tplants.2012.06.001 PMC3466422

[B116] Thelen J. J. Peck S. C. (2007). Quantitative proteomics in plants: choices in abundance. *Plant Cell* 19 3339–3346 18055608 10.1105/tpc.107.053991 PMC2174896

[B117] van Bentem S. D. Roitinger E. Anrather D. Csaszar E. Hirt H. (2006). Phosphoproteomics as a tool to unravel plant regulatory mechanisms. *Physiol. Plant.* 126 110–119

[B118] van Wijk K. J. (2004). Plastid proteomics. *Plant Physiol. Biochem.* 42 963–977 15707834 10.1016/j.plaphy.2004.10.015

[B119] Washburn M. P. Wolters D. Yates J. R. (2001). Large-scale analysis of the yeast proteome by multidimensional protein identification technology. *Nat. Biotechnol.* 19 242–247 11231557 10.1038/85686

[B120] Wendelboe-Nelson C. Morris P. C. (2012). Proteins linked to drought tolerance revealed by DIGE analysis of drought resistant and susceptible barley varieties. *Proteomics* 12 3374–3385 23001927 10.1002/pmic.201200154

[B121] Wienkoop S. Larrainzar E. Niemann M. Gonzalez E. M. Lehmann U. Weckwerth W. (2006). Stable isotope-free quantitative shotgun proteomics combined with sample pattern recognition for rapid diagnostics. *J. Sep. Sci.* 29 2793–2801 17305241 10.1002/jssc.200600290

[B122] Wiese S. Reidegeld K. A. Meyer H. E. Warscheid B. (2007). Protein labeling by iTRAQ: A new tool for quantitative mass spectrometry in proteome research. *Proteomics* 7 340–350 17177251 10.1002/pmic.200600422

[B123] Wisniewski J. R. Zougman A. Nagaraj N. Mann M. (2009). Universal sample preparation method for proteome analysis. *Nat. Methods* 6 359–362 19377485 10.1038/nmeth.1322

[B124] Wortman J. R. Haas B. J. Hannick L. I. Smith R. K. Maiti R. Ronning C. M. (2003). Annotation of the *Arabidopsis* genome. *Plant Physiol.* 132 461–468 12805579 10.1104/pp.103.022251 PMC166989

[B125] Xiao Z. Conrads T. P. Lucas D. A. Janini G. M. Schaefer C. F. Buetow K. H. (2004). Direct ampholyte-free liquid-phase isoelectric peptide focusing: application to the human serum proteome. *Electrophoresis* 25 128–133 14730577 10.1002/elps.200305700

[B126] Yeung Y. G. Stanley E. R. (2010). Rapid detergent removal from peptide samples with ethyl acetate for mass spectrometry analysis. *Curr. Protoc. Protein Sci.* Chapter 16: Unit 16.12 10.1002/0471140864.ps1612s59 PMC2852680 20155730

[B127] Ytterberg A. J. Jensen O. N. (2010). Modification-specific proteomics in plant biology. *J. Proteomics* 73 2249–2266 20541636 10.1016/j.jprot.2010.06.002

[B128] Zybailov B. Rutschow H. Friso G. Rudella A. Emanuelsson O. Sun Q. (2008). Sorting signals, N-terminal modifications and abundance of the chloroplast proteome. *PLoS ONE* 3: e1994 10.1371/journal.pone.0001994 PMC2291561 18431481

